# Intraperitoneal bladder rupture mimicking acute renal failure

**DOI:** 10.4103/0971-4065.41286

**Published:** 2008-01

**Authors:** K. G. Arun, Salahauddin, V. Leela, J. Noel, K. Venkatesh, S. Ramakrishnan, R. Dilip

**Affiliations:** Department of Nephrology, NU Trust, Bangalore, India

**Keywords:** Acute renal failure, bladder rupture

## Abstract

We describe a patient who presented with acute onset of abdominal pain, oliguria, gross hematuria and uremia. Further examination revealed spontaneous intraperitoneal rupture of the urinary bladder. Upon repair of the rupture, his biochemistry normalized within 24 h. Acute renal failure is characterized by an abrupt decline in glomerular filtration rate (GFR), biochemically reflected by elevation in serum creatinine level. It usually displays multifactorial etiology and can be classified as prerenal, renal and postrenal. In this case report, we describe a situation in which the increase in serum creatinine level was not related to any of these factors and was associated with normal GFR and absorption of urine across the peritoneal membrane following spontaneous rupture of bladder.

Sudden elevation in serum creatinine is almost always the result of abrupt decline in renal function (acute renal failure). Rarely it can be associated with normal GFR and be the result of absorption of urine across the peritoneal membrane, as in this case report.

## Case Report

A 32-year-old male presented with acute abdominal pain, reduced urine output and red urine since three days. No fever, vomiting or bowel or bladder symptoms were observed. The pain in the abdomen was diffuse and more as discomfort, which started following a jerk while the patient was traveling in a bus. No prior history of diabetes, hypertension or renal disease was reported.

Investigations conducted in a local hospital had revealed numerous red blood corpuscles (RBCs) and white blood corpuscles (WBCs) in urine sediment and 4 + proteinuria. Ultrasound showed moderate ascites. Plain computed tomography (CT) of the abdomen showed hepatomegaly with ascites. Serum creatinine measured the following day was high (4.0 mg/dl); hence, an opinion from a nephrologist was sought.

Upon examination, he was anxious and hemodynamically stable. The lungs were clear and abdominal distension and ascites was present. Pedal edema was absent. Following are the reports of the investigation carried out: hemoglobin, 17.6 g/dl; total leucocyte count, 20100 cells/m^3^; differential count polymorphs, 79%; lymphocytes, 17%; eosinophils and monocytes, 2% each; urine showed 3 + protein; 6-8 WBCs and numerous RBCs; arterial blood gas analysis pH, 7.31; bicarbonate, 10.8 meq/l; pCo_2_, 22.2 mmHg; blood urea nitrogen (BUN), 60 mg/dl; creatinine, 5.9 mg/dl; sodium, 131 meq/l; potassium, 5.1 meq/l; calcium, 8.6 mg/dl; phosphorus, 6.7 mg/dl; uric acid, 10.2 mg/dl; total bilirubin, 0.3 mg/dl; protein, 8.0 g/dl; albumin, 4.2 g/dl; serum glutamic-oxaloacetic transaminase (SGOT), 38 u/l; serum glutamic-pyruvic transaminase (SGPT), 25 u/l; alkaline phosphatase (ALP), 96 u/l.

**Fig. 1:**
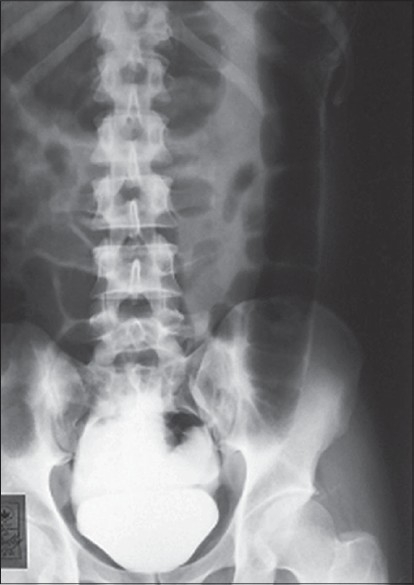
Cystogram showing leakage of the contrast into the peritoneal cavity

We suspected intraperitoneal rupture of the urinary bladder because of the abrupt onset of symptoms. We performed catheterization of the urinary bladder, draining 3.5 l of hemorrhagic fluid. Abdominal distension began to regress and the patient started feeling relieved after this drainage. Cystogram showed leakage of the contrast into the peritoneal cavity [Fig. 1]. Ascitic fluid creatinine and urea nitrogen approximated the values that are usually observed in urine samples rather than in blood (86 mg/dl and 425 mg/dl, respectively).

Following this confirmation, the patient underwent emergency laparotomy. A 5-mm tear was found in the dome of bladder, which was repaired in two layers. He was maintained on indwelling urethral and suprapubic Foley catheters. Within 24 h, the biochemical abnormalities in the patient improved to normal.

## Discussion

Bladder ruptures can be extraperitoneal or intraperitoneal, depending on the site of injury. A mixed picture occurs in a small percentage of cases following abdominal trauma associated with multiple organ injuries. Intraperitoneal bladder rupture is usually associated with blunt abdominal trauma and involves the dome of the bladder in contrast to extraperitoneal rupture, which is usually associated with pelvic fractures and is located in the lateral walls.

Spontaneous rupture of the bladder is very rare, although an overdistended bladder can rupture with surprisingly minor and unnoticed events such as when an individual is under the influence of alcohol.^1^ In the instance presented here, the gentleman who denied alcohol intake prior to this episode had been postponing urination for social reasons for hours; further, with a jolt during bus travel, the whole bladder ruptured intraperitoneally.

The dome of the bladder is the area that is least supported and is the only peritoneum-covered portion in the adult bladder. With the increase in intravesical pressure, the muscle bundles gets separated widely and the entire bladder becomes relatively thin, offering little resistance to rupture due to further increase in the intravesical pressure. An intravesical pressure of more than 300 cm of water is required for the rupture of a normal bladder. Nearly always an underlying pathology that weakens the bladder wall is present mostly in the form of previous bladder surgery; pelvic radiotherapy or bladder tumor and bladder rupture may not be evident during the initial evaluation. In our case, we were unable to detect any underlying bladder pathology.

A cystogram is the diagnostic procedure of choice. With intraperitoneal rupture, a cystogram shows a contrast collecting within the peritoneal cavity, outlining the bowel loops as cylindrical filling defects and the formation of an hourglass configuration where the waist of the hourglass is the point of rupture on a supine film.

Biochemical features of renal failure following intraperitoneal rupture of the urinary bladder are well elucidated and result mainly from the peritoneal diffusion of various solutes excreted in the urine toward the concentration gradient (also termed as reverse autodialysis). The longer the time to presentation, the more severe will be the biochemical abnormalities. Upon suturing the bladder rupture, the biochemical values fall down dramatically.^2^ This is a situation in which renal function is normal in the presence of elevated serum creatinine level and the same can be demonstrated by intravenous urogram (IVU) and radionuclide scans and considered as renal pseudofailure.^3^,^4^ While surgery is required for larger tears, patients with smaller perforations can be managed conservatively.

In patients with sudden onset of abdominal pain, hematuria and the biochemical features of renal failure (elevated serum urea, creatinine and potassium, decreased serum sodium and CO_2_ content), the clinician should suspect an intraperitoneal rupture of the urinary bladder.

## References

[1] Scott DJ, Gibson P, Winney RJ, Fearon KC (1999). An unusual cause of abdominal pain following an alcoholic bout. Postgrad Med J.

[2] Heyns CF, Rimington PD (1987). Intraperitoneal rupture of the bladder causing the biochemical features of renal failure. Br J Urol.

[3] Delfino VD, Jaqueto M, de Freitas Rodrigues MA, Moselin AJ (2006). A man with a serum creatinine near 10 mg/dl with an intravenous pyelogram showing normally functioning kidneys. Nephrol Dial Transplant.

[4] Wystrychowski A, Nowicki M, Kokot F (1996). Hyponatraemic renal pseudofailure-don't forget the possibility of uroperitoneum. Nephrol Dial Transplant.

